# Case Report: High-Gamma Oscillations on an Ictal Electroencephalogram in a Newborn Patient With Hypoxic–Ischemic Encephalopathy

**DOI:** 10.3389/fped.2021.679771

**Published:** 2021-10-01

**Authors:** Akihito Takeuchi, Takushi Inoue, Makoto Nakamura, Misao Kageyama, Tomoyuki Akiyama, Katsuhiro Kobayashi

**Affiliations:** ^1^Division of Neonatology, National Hospital Organization Okayama Medical Center, Okayama, Japan; ^2^Division of Child Neurology, National Hospital Organization Okayama Medical Center, Okayama, Japan; ^3^Department of Child Neurology, Okayama University Graduate School of Medicine, Dentistry and Pharmaceutical Science, Okayama, Japan

**Keywords:** neonatal seizure, fast oscillations, gamma, hypoxic-ischemia encephalopathy, ictal EEG

## Abstract

Fast oscillations (FOs) >40 Hz in electroencephalograms (EEGs) are associated with ictogenesis and epileptogenesis in adults and children with epilepsy. However, only a few previous studies showed FOs in neonates. Reported frequencies of such neonatal FOs were in the low-gamma (<60 Hz) band and, therefore, they were not high compared to those in pediatric patients. We herein report a newborn patient with severe hypoxic–ischemic encephalopathy (HIE), who showed pathological FOs with a frequency in the high-gamma band. She was born at a gestational age of 39 weeks 4 days by emergency cesarean section because of non-reassuring fetal status. She had focal motor seizures involving unilateral upper and lower limbs lasting for tens of seconds on days 0, 1, 4, 5, 8, and 9 and subclinical seizures on days 4–11. Phenobarbital (PB) was intravenously administered on days 0, 2, 4, 5, and 6. We found FOs that were superimposed on the ictal delta activities using visual inspection and time–frequency analysis on 8–11 days of age. Among them, we detected high-gamma (71.4–100 Hz) oscillations that appeared to be temporally independent of low-gamma activities in the ictal EEG on 11 days of age. To the best of our knowledge, this is one of the earliest reports showing pathological FOs with a frequency of >60 Hz in the high-gamma band in human neonatal seizures, which were previously observed in animal studies. Further studies are needed to elucidate the pathophysiology of ictal FOs in neonatal seizures.

## Introduction

High-frequency oscillations (HFOs) ≥80 Hz on electroencephalograms (EEG) and fast oscillations (FOs) ≥40 Hz on scalp EEGs are associated with ictogenesis and epileptogenesis in adults and children with epilepsy (1, 2). However, only a few previous studies showed FOs in neonates with seizures (3–5). Furthermore, the reported frequencies of neonatal FOs were mostly in the low-gamma (<60 Hz) band (4–6) and were, therefore, not high compared to those recorded from pediatric patients with epilepsy (1, 6, 7). The single exception is that Noorlag et al. very recently reported three newborn patients with ictal FOs ≧80Hz (8). However, the pathological meaning of FOs in newborn patients remains unclear.

## Case Report

The patient was a newborn female infant with severe hypoxic–ischemic encephalopathy (HIE). She was born at a gestational age of 39 weeks 4 days by an emergency cesarean section because of non-reassuring fetal status. Her Apgar score was 3 and 5 at 1 min and 5 min, respectively. She was intubated soon after birth and underwent intensive treatment. Brain sonography showed diffuse brain edema and abnormally increased echogenicity, and EEG showed a burst suppression pattern on admission. However, brain hypothermia therapy could not be performed because of severe persistent pulmonary hypertension of the newborn. Brain magnetic resonance imaging (MRI) studies subsequently disclosed diffusely decreased apparent diffusion coefficient values at 2 days of age and multi-cystic encephalomalacia at 17 days of age. She depended on tube feeding that began in the neonatal period. Gastrostomy and tracheostomy were performed at 2 and 3 years of age, respectively. She has profound developmental delay without speech and is bed-ridden with severe tetraplegia until the present age of 8 years. Although she has shown involuntary movements (myoclonus) since infancy, she had no epileptic seizures after the neonatal period.

For her neonatal seizures that were observed from the first day of life to 13 days of age, we recorded a long-term eight-channel video-EEG with a sampling frequency at 500 Hz using a Nihon–Kohden Neurofax (EEG-1250, Nihon–Kohden, Tokyo, Japan). Electroencephalograms monitoring was done in an unshielded neonatal intensive care unit and no special shielding devices were used. We used passive Ag/AgCl electrodes and made an effort to maintain an electrode impedance of less than 10 kΩ during recording. She had focal motor seizures involving unilateral upper and lower limbs lasting for tens of seconds on days 0, 1, 4, 5, 8, and 9, and subclinical seizures on days 4–11. Phenobarbital (PB) was intravenously administered on days 0 (5 mg/kg), 2 (4 mg/kg), 4 (10 mg/kg), 5 (15 mg/kg), and 6 (10 mg/kg). Serum concentration of PB was 39.4 μg/ml at 6 days of age.

**Figure 1:**
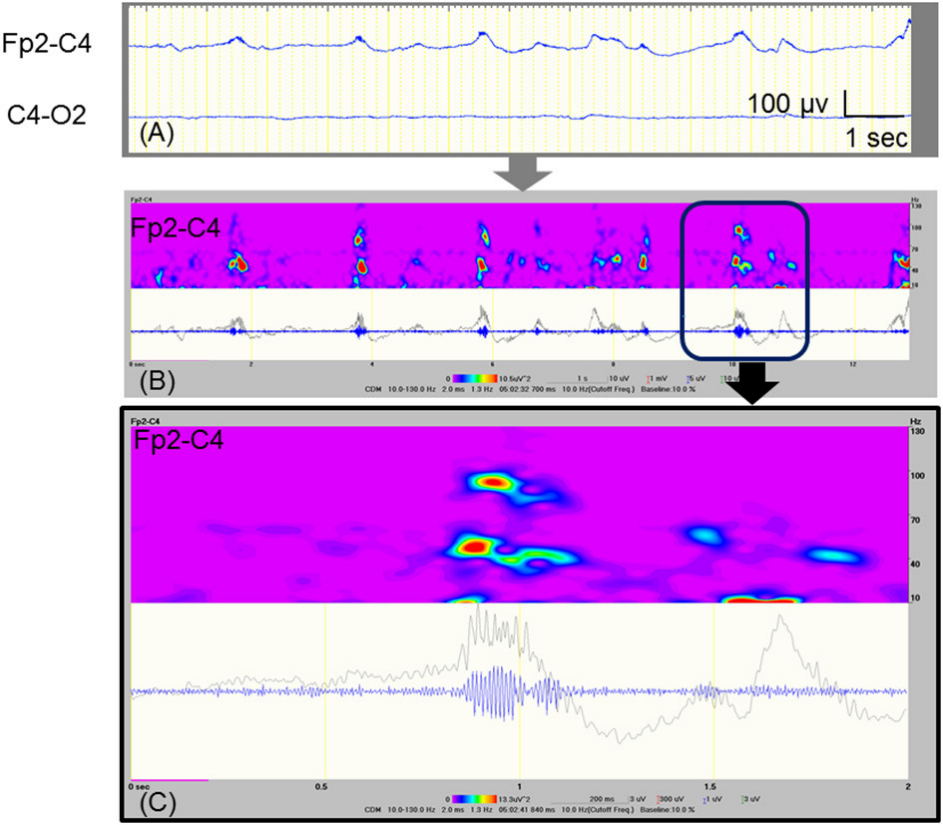
Ictal EEG with FOs that were recorded at 11 days of age. **(A)** Raw EEG traces, which are analyzed as shown in **(B)**. **(B)** Time–frequency analysis of the EEG data at Fp2-C4 that is shown in **(A)**, using the Complex Demodulation Method. The gray trace is unfiltered, and the blue trace is applied with a high pass filter at 80 Hz. The area indicated by the square corresponds to expanded data shown in **(C)**. **(C)** The detected high-gamma (71.4–100 Hz) activities appear to be temporally independent of the low-gamma peaks.

At 10 and 11 days of age, we found obvious fast activities superimposed on the ictal delta activities using visual inspection (Figure 1A). Therefore, we examined all artifact-free ictal EEGs through temporal expansion of traces and time-frequency spectral analysis. The conventional EEG traces were initially reviewed to identify the EEG seizures using a time constant of 0.3 s and a low-pass filter at 120 Hz. The traces of EEG seizures were then temporally expanded with a time constant of 0.03 s without any low-pass filter to study the details of the FOs. We built time–frequency spectra for EEG seizures by applying the Complex Demodulation Method (Nihon–Kohden Neuroworkbench ver. 07-03: frequency range, 10–150 Hz) to the artifact-free clear EEG data. Complex demodulation is a method to detect frequency components in the time course of EEG signals by computing the instantaneous frequency and momentary power (9). Table 1 shows daily information including the number of clinical seizures, clinical seizures with FOs (>40 Hz), subclinical seizures, subclinical seizures with FOs, and total duration of artifact-free EEG recording. We found FOs superimposed over delta waves in one clinical seizure and 42 subclinical focal seizures recorded at 8–11 days of age. Among them, we detected high-gamma (71.4–100 Hz) oscillations that had spectral peaks separate from those of low-gamma activities in the ictal EEG on 11 days of age (Figures 1B,C).

**Table 1:** Numbers of seizures in artifact free EEG records.

**Days after birth**	**Clinical Sz**	**Clinical Sz with FO**	**Subclinical Sz**	**Subclinical Sz with FO**	**Total duration of artifact-free EEG recording**
0	6	0	0	0	2:55
1	1	0	0	0	15:20
2	0	0	0	0	1:28
3	0	0	0	0	2:28
4	68	0	5	0	9:35
5	0	0	1	0	4:11
6	0	0	20	0	5:22
7	0	0	3	0	3:48
8	0	0	4	2	1:51
9	3	1	29	6	5:34
10	0	0	17	13	3:01
11	0	0	24	21	6:24
12	0	0	0	0	1:25
13	0	0	0	0	8:40

*Sz, seizure, FO, fast oscillation (>40 Hz), EEG, electroencephalogram*.

## Discussion

For pathological FOs in neonates, Yamazaki et al. reported abnormal FOs at around 50 Hz (up to 57 Hz) that were associated with burst patterns on the inter-ictal EEG in newborn patients with hemimegalencephaly (3). These pathological FOs had frequencies that were higher than brushes, which are well-known physiological oscillations with a frequency ranging from 15 to 20 Hz in neonates (10). For the ictal EEG, Nagarajan et al. reported neonatal seizure-associated FOs (>30 Hz) in 17 out of 42 babies with EEG-confirmed seizures with various etiologies (4). They showed that FOs that were superimposed on seizure activity in the delta band appeared were similar to FOs in the present patient. They described that the ictal FOs were mostly between 35 and 55 Hz without disclosure of the detailed data. However, these studies had the limitation of low sampling rates (e.g., 256 Hz) for the EEG recording, which may have caused signal loss above 70–80 Hz due to anti-aliasing filtering. Fast activity with attenuated background activity at the onset of seizures in neonatal patients with *KCNQ2*-related epilepsies (*KCNQ2* encephalopathy and benign familial neonatal epilepsy) has been reported (5, 11), but the highest frequency of such fast activity is unknown due to a lack of frequency analysis. Just recently, Noorlag et al. studied high-frequency (≧80Hz) EEG oscillations among 16 patients with neonatal seizures using a very high EEG sampling rate, and they found two term infants and one preterm infant (19% of patients) with ictal FOs during 13 seizures (7% of the 175 seizures in the 16 patients) (8). Their report and ours showed that ictal FO ≧60 Hz can be generated by the neonatal brain, but the occurrence does not seem to be high.

Pathological FOs in adults are thought to be related to an abnormal glutamatergic network, which consists of recurrent excitatory synaptic transmission and pyramidal axo-axonic gap junctions (12). However, pathological gamma oscillations in the early developing brain seem to be related to γ-aminobutyric acid (GABA) interneurons and possibly electrical gap junctions between interneurons (12). γ-Aminobutyric acid neurons have excitatory action in early infancy as a result of high expression of the chloride loader NKCC1 and low expression of the chloride extruder KCC2 (13). According to a previous study (14) in rats, high-frequency bursts of action potentials in the gamma range in premature neurons (tissue from a focus of secondary epileptic activity in the P7 rat brain) induced by electrical stimulation were completely blocked by a GABA receptor antagonist (15). However, it was suggested that GABAergic neurons alone cannot generate pathologic FOs >60 Hz (high-gamma range) in the premature brain and that the maturation of synapses using glutamate is needed to generate pathologic FOs in the high-gamma range (12). Frequencies of FOs that were shown in a previous study were in the 40–60 Hz band in rats before P6, which is equivalent to human preterm infants (15). This might be related to the insufficient glutamatergic synapse density level (15). After P6, which is equivalent to term gestational age and thereafter in humans, maturation of GABAergic and glutamatergic synapses and particularly N-methyl-D-aspartate receptors reaches a sufficient level of organization to drive pathologic FOs >60 Hz (12, 15). Therefore, high-gamma activity observed in the present patient suggested that both excitatory GABA neurons and partly pyramidal neurons might generate ictal FOs even in the human term newborn with HIE. As described above, it is thought that high frequency ictal FOs are not seen in rats corresponding to preterm human infants. However, Noorlag et al. reported a case of a preterm infant born at 31 weeks of gestational age with ictal FOs ≧80 Hz (8), suggesting that ictal FOs may appear even before 37 weeks in humans.

The pathological significance of ictal FOs has been discussed in previous reports. Nagarajan et al. studied 42 cases of neonatal seizures; however, they could not find clear associations between FO <60 Hz and PB administration, background EEG, the presence of seizure symptoms, neuroimaging abnormalities, mortality, neurodevelopmental abnormalities, or post neonatal seizures (4). On the other hand, Noorlag et al. stated that the pathological meaning of FO ≧80 Hz was not known because of the small number of cases (8). Previous studies on ictal FO in neonates, including our report, have not yet clarified the pathological significance and clinical value of FO, and the accumulation of future cases will be very important.

There was one neonate with HIE in the report by Noorlag et al. (8). In that case, ictal EEGs were recorded on day 5 and day 15, and FOs were seen only in the seizure on day 15 (8). Interestingly, our case also showed ictal FOs only after 10 days of age, and the similarity is that the FOs appeared in the latter half of the EEG monitoring period. The strength of our report is the use of very long-term EEG recordings from birth to day 13. We believe this is a valuable report for considering the time course of FO occurrence, although only a small portion (72 h in total) of the long-term (14 days) EEG recordings could be analyzed due to artifacts. On the other hand, the obvious limitation is that results of this EEG study have been observed only in one newborn patient. Therefore, the results and conclusions cannot be extended to all neonates with HIE.

To the best of our knowledge, this is one of the earliest reports that shows the presence of pathological FOs with a frequency of >60 Hz in the high-gamma band in human neonatal seizures, which were previously observed in animal studies. Further research is needed to elucidate the pathophysiology and clinical value of ictal FOs in neonatal seizures.

## Data Availability Statement

The raw data supporting the conclusions of this article will be made available by the authors, without undue reservation.

## Ethics Statement

Ethical review and approval was not required for the study on human participants in accordance with the local legislation and institutional requirements. Written informed consent from the participants' legal guardian/next of kin was not required to participate in this study in accordance with the national legislation and the institutional requirements. Written informed consent was obtained from the minor(s)' legal guardian/next of kin for the publication of any potentially identifiable images or data included in this article.

## Author Contributions

AT conceptualized and designed the study, carried out the initial analyses, drafted the initial manuscript, and reviewed and revised the manuscript. TI, MN, and MK designed the study, reviewed, and revised the manuscript. KK and TA designed the study, carried out the analyses, critically reviewed, and revised the manuscript for important intellectual content. All authors approved the final manuscript as submitted and agree to be accountable for all aspects of the work.

## Funding

KK was supported by Grants-in-Aid from the Ministry of Education, Culture, Sports, Science and Technology, Japan [MEXT KAKENHI Grant Number 15H05874 (Non-linear Neuro-oscillology)] and by Health and Labour Research Grants from the Ministry of Health, Labour and Welfare, Japan (H26-nanchitou-ippan-051, and H29-nanchitou-ippan-010).

## Conflict of Interest

The authors declare that the research was conducted in the absence of any commercial or financial relationships that could be construed as a potential conflict of interest.

## Publisher's Note

All claims expressed in this article are solely those of the authors and do not necessarily represent those of their affiliated organizations, or those of the publisher, the editors and the reviewers. Any product that may be evaluated in this article, or claim that may be made by its manufacturer, is not guaranteed or endorsed by the publisher.
